# Antimicrobial resistance and genomic analysis of staphylococci isolated from livestock and farm attendants in Northern Ghana

**DOI:** 10.1186/s12866-022-02589-9

**Published:** 2022-07-21

**Authors:** Beverly Egyir, Esther Dsani, Christian Owusu-Nyantakyi, Grebstad Rabbi Amuasi, Felicia Amoa Owusu, Emmanuel Allegye-Cudjoe, Kennedy Kwasi Addo

**Affiliations:** 1 Department of Bacteriology, Noguchi Memorial Institute for Medical Research, University of Ghana, Accra, Ghana; 2 Regional Veterinary Laboratory, Ho, Volta Region Ghana; 3 Central Veterinary Laboratory, Pong-Tamale, Northern Region, Ghana

**Keywords:** Staphylococci, Antimicrobial resistance, Multi-drug resistance, WGS, Ghana

## Abstract

**Background:**

The emergence of antimicrobial resistant bacteria in food producing animals is of growing concern to food safety and health. Staphylococci are common inhabitants of skin and mucous membranes in humans and animals. Infections involving antibiotic resistant staphylococci are associated with increased morbidity and mortality, with notable economic consequences. Livestock farms may enable cross-species transfer of antibiotic resistant staphylococci. The aim of the study was to investigate antimicrobial resistance patterns of staphylococci isolated from livestock and farm attendants in Northern Ghana using phenotypic and genotypic methods. Antimicrobial susceptibility testing was performed on staphylococci recovered from livestock and farm attendants and isolates resistant to cefoxitin were investigated using whole genome sequencing.

**Results:**

One hundred and fifty-two staphylococci comprising *S. sciuri* (80%; *n* = 121), *S. simulans* (5%; *n* = 8), *S. epidermidis* (4%; *n* = 6), *S. chromogens* (3%; *n* = 4), *S. aureus* (2%; *n* = 3), *S. haemolyticus* (1%; *n* = 2), *S. xylosus* (1%; *n* = 2), *S. cohnii* (1%; *n* = 2), *S. condimenti* (1%; *n* = 2), *S. hominis* (1%; *n* = 1) and *S. arlettae* (1%; *n* = 1) were identified. The isolates showed resistance to penicillin (89%; n = 135), clindamycin (67%; n = 102), cefoxitin (19%; *n* = 29), tetracycline (15%; *n* = 22) and erythromycin (11%; *n* = 16) but showed high susceptibility to gentamicin (96%; *n* = 146), sulphamethoxazole/trimethoprim (98%; *n* = 149) and rifampicin (99%; *n* = 151). All staphylococci were susceptible to linezolid and amikacin. Carriage of multiple resistance genes was common among the staphylococcal isolates. Genome sequencing of methicillin (cefoxitin) resistant staphylococci (MRS) isolates revealed majority of *S. sciuri* (93%, *n* = 27) carrying *mecA1* (which encodes for beta-lactam resistance) and the *sal(A)* gene, responsible for resistance to lincosamide and streptogramin. Most of the MRS isolates were recovered from livestock.

**Conclusion:**

The study provides insights into the genomic content of MRS from farm attendants and livestock in Ghana and highlights the importance of using whole-genome sequencing to investigate such opportunistic pathogens. The finding of multi-drug resistant staphylococci such as *S. sciuri* carrying multiple resistant genes is of public health concern as they could pose a challenge for treatment of life-threatening infections that they may cause.

## Background

Staphylococci are the most common bacteria found on the skin and mucous membranes of mammals [1]. Bacteria of this genus are usually commensal organisms and can be divided into two groups based on their ability to produce the enzyme coagulase [2]. Pathogenic infections may result from colonization with staphylococci if primary skin barriers are compromised due to trauma [3]. Among the species producing coagulase, *Staphylococcus aureus* is of primary importance to human and animal health. *S. aureus* is a notable cause of mastitis in livestock and has been associated with bacteremia, skin and soft tissue infections in humans [4, 5].

The less pathogenic group of coagulase negative staphylococci (CoNS) have garnered interest in recent times due to their increasing role in the occurrence of opportunistic infections [6]. CoNS-related infections in humans have been associated with the presence of foreign bodies and immunosuppressive states of patients [7]. *Staphylococcus epidermidis* falls within the category of CoNS and is the most common cause of foreign body related blood stream infections in humans [8]. *S. epidermidis* and *S. chromogens* have been isolated in subclinical and clinical mastitis cases in cattle [9]. CoNS are known carriers of transferable genetic elements that contribute to the survival of some strains of *S. aureus* and are often cited as reservoirs of resistance genes [10].

The emergence of antimicrobial resistance (AMR) in staphylococci, though partly dependent on innate microbial characteristics, is mainly driven by antimicrobial use [11, 12] *S. aureus* are known to adapt and gain resistance to nearly all antibiotics used to treat it. Concurrent resistance to non-β-lactam agents such as quinolones, tetracyclines, aminoglycosides, macrolides and lincosamides are increasingly reported among methicillin resistant strains of staphylococci, further diminishing the treatment options available for infected humans or animals [13]. Methicillin resistance is attributed to the production of a transpeptidase (PBP2a), encoded by the *mecA* gene within the staphylococcal cassette chromosome [14]. Methicillin resistant *S. aureus* (MRSA) infections often result in increased morbidity and an increase in health care associated costs [15].

Mounting evidence suggests that the development of resistance in commensal pathogens of livestock origin contribute to the persistence of carriage of these resistant organisms in humans. Antimicrobials are used routinely in livestock production and its misuse in livestock farming often leads to emergence of resistance due to selective pressure on microbes like staphylococci exposed to antibiotics [16]. Colonized livestock may spread resistant strains directly to humans or indirectly through the food chain [17]. The detection of MRSA in humans that have been linked to animals has heightened concerns of its ability to be transferred among species [18]. Livestock farmers, veterinarians, wool sorters, meat hygiene inspectors and people who frequently visit livestock farms have been found to be at increased risk for MRSA colonization [19].

The presence of multidrug resistant strains of staphylococci in livestock have implications for food safety and contribute to the global challenge of AMR [20]. Knowledge on carriage rates of resistant strains of CoNS in livestock is scarce in Ghana; such information is necessary to inform antimicrobial policies and improve integrated surveillance on antimicrobial resistance. Findings from antimicrobial susceptibility testing of bacteria species such as CoNS offer vital information for surveillance but are limited when it comes detection of clones, resistance and virulence of bacteria pathogens. Genomic sequencing on the other hand, provides massive information for characterizing bacteria species [21] including staphylococci. This study therefore sought to characterize staphylococci recovered from livestock and farm attendants in the Northern part of Ghana using phenotypic and genotypic methods.

## Results

### Farm attendant characteristics

Majority of the farm attendants were male (89%). The age of farm attendants ranged from 14 to 58 years. Daily participation in livestock rearing activities such as feeding, grazing and handling was reported by all 19 respondents. Most of the livestock attendants worked primarily on only one type of livestock (84%). Three attendants worked with more than one livestock type. Most of the farm attendants lived on the farm (83%) and had been working on the farm for at least eight years (78%).

### Nasal carriage of staphylococci

Of the 311 nasal swabs collected from livestock and 19 samples collected from farm attendants, 152 staphylococcal isolates were recovered. Staphylococci were obtained in close to half of livestock samples (45%; *n* = 141) and in most farm attendant samples (58%; *n* = 11). Most of the isolates (98%; *n* = 149) were CoNS, with three isolates (2%) identified as *S. aureus*. Ten different CoNS species were confirmed, with *S. sciuri* being the most prevalent (80%; *n* = 121). Others include *S. simulans* (5%; *n* = 8), *S. epidermidis* (4%; *n* = 6), *S. chromogens* (3%; *n* = 4), *S. haemolyticus* (1%; *n* = 2), *S. xylosus* (1%; *n* = 2) *S. cohnii* (1%; *n* = 2), *S. condimenti* (1%; *n* = 2), *S. hominis* (1%; *n*

 = 1) and *S. arlettae* (1%; *n* = 1).

*S. sciuri* was found in nasal swabs from all livestock types in this study. *S. epidermidis* was isolated only from nasal swabs obtained from farm attendants*. S. haemolyticus* was isolated from human and sheep samples. *S. condimenti* was found only in pigs, whilst *S. hominis* and *S. arlettae* were isolated only in sheep*.* Six different species of staphylococci were found in samples from goats and consisted of *S. sciuri, S. simulans, S. aureus, S. xylosus, S. chromogens and S. cohnii* (Table 1).

**Table 1:** Nasal carriage of *Staphylococci* among farm attendants and livestock, 2018

Parameter	Humans	Cattle	Sheep	Goat	Pig	Total
No. of isolates	11	19	71	32	19	152
Staphylococcal species						
* S. epidermidis*	6	-	-	-	-	6
* S. haemolyticus*	1	-	1	-	-	2
* S. xylosus*	1	-	-	1	-	2
* S. sciuri*	3	18	65	21	14	121
* S. aureus*	-	-	-	3	-	3
* S. simulans*	-	-	2	3	3	8
* S. chromogens*	-	1	1	2	-	4
* S. cohnii*	-	-	-	2	-	2
* S. hominis*	-	-	1	-	-	1
* S. arlettae*	-	-	1	-	-	1
* S. condimenti*	-	-	-	-	2	2

### Antimicrobial resistance in staphylococcal isolates

Isolates from farm attendants were mainly resistant to penicillin (100%; *n* = 11), tetracycline (55%; *n* = 6), cefoxitin (27%; *n* = 3), clindamycin (36%; *n* = 4), sulphamethoxazole/trimethoprim (27%; *n* = 3), erythromycin (18%; *n* = 2) and gentamycin (18%; *n* = 2). All staphylococcal isolates from farm attendants were susceptible to linezolid, amikacin and rifampicin. Isolates obtained from livestock were mainly resistant to penicillin (88%; *n* = 124), clindamycin (70%; *n* = 98), cefoxitin (18%; *n* = 26), tetracycline (11%; *n* = 16) and erythromycin (10%; *n* = 14) (Table 2). Among isolates from livestock, resistance to gentamicin and rifampicin was less prevalent (3%; *n* = 4 and 1%; *n* = 1). All cefoxitin resistant isolates (19%; *n* = 29) were susceptible to vancomycin.

**Table 2:** Antimicrobial resistance of staphylococci isolated from livestock and farm attendants, 2018

Antimicrobial agent	Livestock *n* = 141 (%)	Farm attendants *n* = 11 (%)	Total *N* = 152(%)
Penicillin	124 (88)	11 (100)	135 (89)
Clindamycin	98 (70)	4 (36)	102 (67)
Cefoxitin	26 (18)	3 (27)	29 (19)
Tetracycline	16 (11)	6 (55)	22 (15)
Erythromycin	14 (10)	2 (18)	16 (11)
Gentamicin	4 (3)	2 (18)	6 (4)
Rifampicin	1 (1)	0 (0)	1 (1)
Sulphamethoxazole-Trimethoprim	0 (0)	3 (27)	3 (2)
Amikacin	0 (0)	0 (0)	0 (0)
Linezolid	0 (0)	0 (0)	0 (0)

The predominant isolates detected, *S. sciuri* were resistant to 6 out of 10 antimicrobials agents tested in all: penicillin (94%; *n* = 114), clindamycin (80%; *n* = 97), cefoxitin (22%; *n* = 27), tetracycline (8%; *n* = 10), erythromycin (8%; *n* = 10) and gentamicin (2%; *n* = 3). *S. epidermidis* isolates were resistant to seven out of 10 antimicrobials with a single isolate exhibiting resistance to six antibiotic agents. Half (50%; *n* = 3) of *S. epidermidis* isolates were resistant to sulphamethoxazole/trimethoprim. Although low numbers of *S. xylosus* isolates were identified, resistance was detected to five out of 10 antibiotics (penicillin, clindamycin, tetracycline, erythromycin and rifampicin). *S. aureus* isolates recovered were resistant to penicillin, tetracycline and erythromycin. *S. chromogens* and *S. cohnii* were susceptible to all tested antibiotic agents except penicillin (Table 3).

**Table 3:** Pattern of antimicrobial resistance of staphylococcal isolates, 2018

		**No.(%) of resistant isolates**							
	No. of isolates	Pen	Clin	Cef	Tet	Ery	Gen	Rif	STX-
*S. sciuri*	121	114 (94)	97 (80)	27 (22)	10 (8)	10 (8)	3 (2)	-	-
*S. epidermidis*	6	6 (100)	1 (17)	1 (17)	5 (83)	2 (33)	1 (17)	-	3 (50)
*S. haemolyticus*	2	2 (100)	1 (50)	1 (50)		1 (50)	1 (50)	-	-
*S. xylosus*	2	2 (100)	2 (100)	-	1 (50)	1 (50)	-	1 (50)	-
*S. simulans*	8	1 (12.5)	-	-	3 (38)	-	-	-	-
*S. chromogens*	4	3 (75)	-	-	-	-	-	-	-
*S. cohnii*	2	2 (100)	-	-	-	-	-	-	-
*S. hominis*	1	1 (100)	-	-	-	-	1 (100)	-	-
*S. arlettae*	1	1 (100)	1 (100)	-	-	1 (100)	-	-	-
*S. condimenti*	2	-	-	-	1 (50)	-	-	-	-
*S. aureus*	3	3 (100)	-	-	2 (67)	1 (33)	-	-	-
Total (N, %)	152	135 (89)	102 (67)	29 (19)	22 (15)	16 (11)	6 (4)	1 (0.7)	3 (2)

Of the 152 staphylococci isolated, 49 (32%) were MDR. Overall, MDR rates were higher in farm attendants (45%; *n* = 5) than in livestock (31%; *n* = 44).

### Genomic analysis of cefoxitin resistant staphylococci

Whole-genome sequencing of the cefoxitin-resistant isolates revealed that all *S. sciuri* possessed *mecA1* gene, while *S. epidermidis* and *S. haemolyticus* harbored *mecA* gene. Tetracycline resistance genes detected were: *tet(K)* (10%, *n* = 3), *tet(L)* (3%, *n* = 1) and *tet(M)* (3%, *n* = 1). The *tet(K)* genes detected were from two goats and one sheep while *tet(M)* and *tet(L)* were detected in an isolate from one farm attendant. Chloramphenicol resistance gene *cat(pC221)* was observed in one *S. sciuri* isolate. *sal(A)* gene responsible for resistance to lincosamide and streptogramin antibiotics was observed in 93% (*n* = 27) of *S. sciuri* isolates. Genes responsible for aminoglycoside (*aadD*), folate pathway (*dfrK; dfrG*), lincosamide and macrolide *(erm(B))*, fosfomycin (*fosB*) and tetracycline (*tet(K), tet(M), tet(L))* resistance were detected in one *S. epidermidis.* Aminoglycoside-resistance gene *aac(6')-aph(2''),* folate pathway antagonistic gene *dfrG*, and beta-lactam gene *blaZ* were detected in the *S. haemolyticus*.

**Fig. 1:**
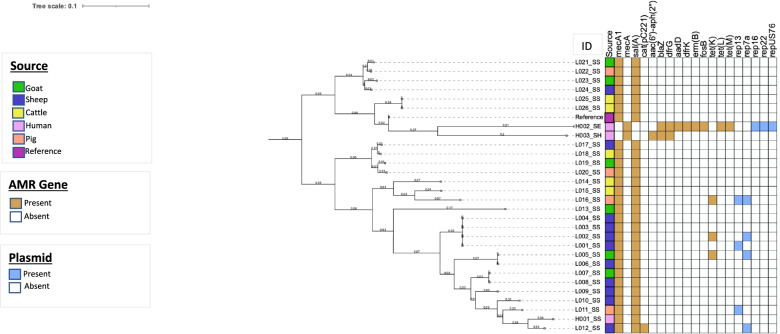
Core Maximum likelihood phylogeny of the Methicillin-Resistant Staphylococci (MRS) isolates (2018). The phylogenetic tree was constructed using CSI-Phylogeny based on core genome SNPs extracted from alignment to reference strain LS483305.1 and visualised using Interactive Tree of Life (iTOL). SS: *Staphylococcus sciuri*, SE: *Staphylococcus epidermidis* SH: *Staphylococcus haemolyticus*

In this study, seven plasmid sequences (rep_13_, rep_7a_, rep_16_, rep_22_, rep_US76_, rep_19_, and rep_20_) were observed in 24% (*n* = 7) of the isolates with rep_7a_ (*n* = 4) predominating. Three of these plasmids (rep_16_, rep_22_, rep_US76_) were detected in an *S. epidermidis* isolate which also harbored the insertion sequence ISSep2. The *S. epidermidis* and *S. haemolyticus* isolates belonged to sequence types 226 and 30 respectively. All *S. sciuri* isolates appear to be novel with unknown sequence types. Core single nucleotide polymorphism (SNP) maximum likelihood tree of the 29 cefoxitin resistant isolates revealed 2 distinct clades. Both clades showed clustering of isolates from all sources. Comparative genomic analysis of the *S. sciuri* isolates obtained from the farm attendants and livestock demonstrated clustering and high-level genetic homogeneity (> 95%), suggesting possible transmissions between hosts (Fig. 1). The complete genome sequences have been deposited at Gene Bank with the following accession numbers: JALGXD000000000-JALGPD000000000.

## Discussion

The findings of this study show that multidrug-resistant staphylococci are prevalent in livestock and farm attendants on the farms sampled. The detection of *S. sciuri, S. aureus*, *S. xylosus*, *S. simulans, S. chromogens*, *S. cohnii*, *S. hominis, S. arlettae and S. condimenti* are consistent with previous reports on prevalence of CoNS in livestock [22, 23]. *S. sciuri* was the most prevalent staphylococci identified in this study (80%) and was isolated in both livestock and farm attendants. Primarily considered a livestock-associated bacterium, *S. sciuri* can be found in large numbers in the farm environment [22]. Though the colonizing population may be low outside the farm environment, they are found to readily adapt and persist in health care settings and thus may pose a threat to human health [23]. *S. sciuri* has been associated with pneumonia and septicemic shock in Grasscutter (*Thryonomys swinderianus*) in Ghana [24]. This pathogen was detected in China as the causative organism for endocarditis [25] and in Thailand, as the agent in a food poisoning outbreak investigation [26].

*S. aureus* was detected in samples from three goats and none contained the *mecA* gene (MRSA). This is in sharp contrast with reports from other geographic areas [9]. Of note, the CoNS that were resistant to cefoxitin and also positive for *mecA1* in this study originated from livestock. Previous studies in Ghana however, found MRSA among farm attendants and none from livestock [27]. MRSA may be more prevalent in intensive farming systems where antimicrobials are used in the production chain more frequently, and in hospital settings due to selective pressure of bacteria in these environments and their presence in colonized inpatients [28]*. S. epidermidis*, *S. haemolyticus*, *and S. xylosus* were frequently detected in samples from farm attendants*.* This concurs with previous reports that identified *S. epidermidis* as the most frequently occurring CoNS found on skin and mucosae in humans [1]. *S. epidermidis* and *S. haemolyticus* have increasingly been associated with nosocomial infections and are described to be highly adaptable to medical devices hence the need to monitor carriage rates in humans [29].

The level of resistance of staphylococci to critically needed antimicrobial agents is a growing public health concern. The overall prevalence of MDR was 32%, with higher rates observed for farm attendants. The high rate of resistance to penicillin observed in this study is consistent with previous reports on staphylococci from both clinical and non-clinical samples in Ghana [27, 30–34] and elsewhere [35, 36] thus, was expected.

The proportion of isolates resistant to tetracycline was higher in isolates from farm attendants (55%) as compared to livestock (11%), and this is in concordance with the rates of tetracycline resistance in staphylococci. More than half of CoNS from livestock attendants (*S. epidermidis*, *S. haemolyticus)* were tetracycline resistant. Tetracycline resistance in all livestock in this study was lower (11%) than was reported in *S. aureus* isolates recovered from livestock (47%) in a previous study in Ghana [27] and from chicken droppings (74%) elsewhere [37]. This observation can be attributed to the frequency of its use in the various farming systems. Tetracycline and its derivatives are often used as part of feed for growth promotion and prophylaxis in intensive poultry farming. Though its use in livestock is widespread, oral preparations are not common.

Resistance to sulphamethoxazole/trimethoprim (STX) was found in *S. epidermidis*, from three farm attendants. STX has been used in the treatment of infections caused by community-acquired *S. aureus;* resistance to this agent among methicillin resistance strains may point to a reduction in its efficacy due to exposure [38]. High susceptibility of isolates to vancomycin, linezolid, amikacin, rifampicin and gentamicin is crucial for the treatment of severe infections in humans. A notable example is the use of vancomycin for the treatment of MRSA infections [39].

Phylogenetic analysis of MRS isolates showed close genetic relatedness between isolates recovered from humans and livestock, suggesting that these pathogens may not be host-specific. The observation could also reflect possible transmission between the different hosts.

The *mec* variants (*mecA* and *mecA1*) found in the MRS have been linked to resistance to beta-lactam antibiotics in staphylococci [40]. Several studies have pointed to *mecA1* detected in *S. sciuri* as an evolutionary ancestor of the *mecA* gene found in MRSA [40–42]. *mecA1* gene is naturally adapted to *S. sciuri;* resistance to beta-lactams due to changes in the promoter region of this gene have been reported [40, 43].

The high levels of resistance to clindamycin among the isolates can be linked to the *sal(A)* gene detected in majority of the *S. sciuri* (93%) isolates. *sal(A*) is located between two housekeeping genes of the core genome of *S. sciuri* subspecies; it encodes for resistance to lincosamide and streptogramin antibiotics [44].

The plasmids detected in this study harbored antibiotic resistance genes. Many resistance determinants are plasmid-mediated, and this has been demonstrated in previous studies on staphylococci [45]. Horizontal transfer of plasmids can occur among staphylococcal strains of different species although data on plasmid distribution in staphylococci are scarce [10, 46]. In this study, seven different plasmid sequences were detected based on the sequences of their rep genes. The most prevalent sequence was rep_7a_, which is similar to studies conducted by Strasheim et al., in which rep_7a_ was detected as one of the most dominant rep genes in *S. aureus* isolates [47].

Co-occurrence of rep_7a_ plasmid with *tet(K)* gene was observed in three isolates. Similarly, the occurrence of rep_7a_ plasmid with *cat(pC221)* was observed in one isolate. This is consistent with reports of strong association between this plasmid, tetracycline and chloramphenicol resistance [48, 49]. Tetracycline resistance genes: *tet(K), (M)* and *(L)* have been reported among staphylococci recovered from clinical and non-clinical samples in African countries including Ghana [27, 33, 35, 50] suggesting that these genes are widely disseminated in these regions.

The potential risks of transfer of plasmid-borne AMR genes from *S. sciuri* to other *Staphylococcus* species was previously reported by Li et al., [51] pointing to the need to routinely monitor AMR gene carriage on plasmids in coagulase negative staphylococci. The presence of AMR genes borne on plasmids in CoNS isolated supports the evidence that CoNS may serve as reservoirs for the spread of AMR [10]. Dissemination of plasmids carrying multiple resistance genes will substantially limit the efficacy of antibiotic agents and urgently warrants surveillance of staphylococci from animal and human sources. Previous studies have shown that plasmid sequence associated with rep_16_ and rep_20_ was prevalent in clinical *S. aureus* and *S. haemolyticus* isolates [52]. rep_13_, rep_16_, rep_19_ rep_22_ and rep_20_ have also been detected in *S. aureus* isolates from different geographic regions with rep_16_ carrying multiple resistance genes [53, 54]. To the best of our knowledge, the co-occurrence of *erm(B), dfrK, aadD* resistance genes and replicon plasmids rep_16_, rep_22_, rep_US76_ in ST 226 *S. epidermidis* has not been reported from farm attendants in previous studies in Ghana. Interestingly, ST 226 *S. epidermidis* was detected in a blood sample of a neonate in Ghana and from hospital and community settings in China [55, 56]; on the other hand, ST30 *S. haemolyticus* found in this study, has also been detected in blood stream and catheter related infections from India, often showing vancomycin heteroresistance [57, 58] thus, confirming invasive characteristics of these CoNS clones.

## Conclusion

The study provides insights into the genomic content of MRS from farm attendants and livestock in Ghana and therefore highlights the importance of using whole-genome sequencing to investigate such opportunistic pathogens. CoNS may serve as reservoirs for transmission of resistant genes to *S. aureus* at the farm level among livestock and farm attendants. The finding of multi-drug resistant CoNS including *S. sciuri* carrying multiple resistant genes is of public health concern as they could pose a challenge for treatment of life-threatening infections that they may cause.

## Materials and methods

### Study sites and sample collection

**Fig. 2:**
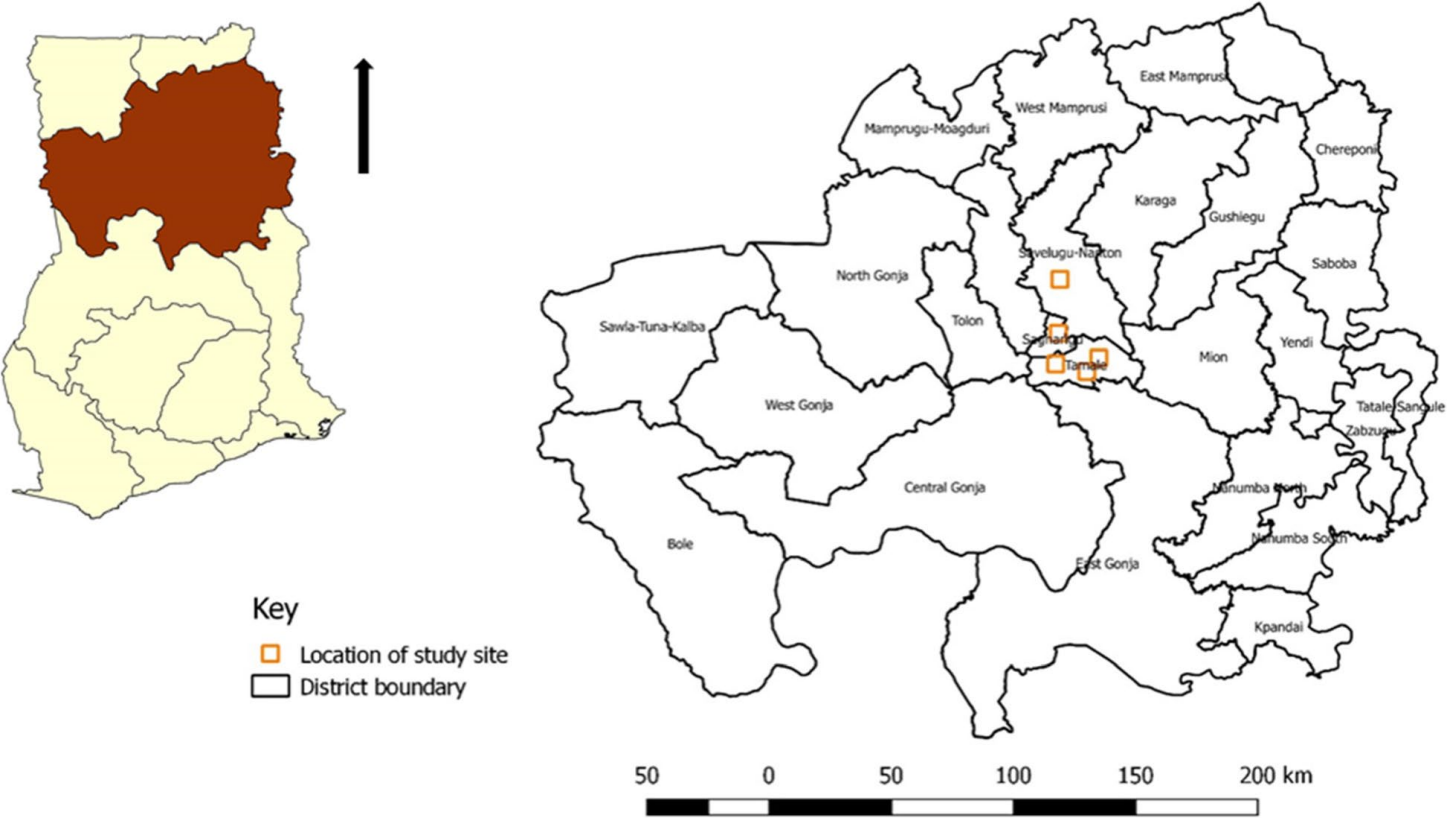
Location of selected livestock farms in the Northern Region of Ghana (2018)

The Northern region is a major hub for livestock production in Ghana, consisting of numerous smallholder and pastoral farming systems [59]. Samples were collected in July 2018 from one multi-species livestock breeding station and four livestock farms in the Northern region of Ghana (Fig. 2). The Livestock breeding station was selected purposively due to its role in the livestock supply chain in the Northern region. The four farms were selected randomly from 2 districts in the region which were integrated with households in four communities. Nasal swabs were obtained from three hundred and eleven (311) livestock and nineteen (19) farm attendants. Livestock on selected farms were selected randomly within their pens or sheds making sure to collect samples from at least five pens on farms with more than five pens. On farms with very few pens (≤ 5) samples were collected from at least three pens.

All nasal swabs were placed in 10 ml of Mueller–Hinton broth (Oxoid Ltd., Basingstoke, UK), supplemented with 6.5% NaCl in sterile tubes and labeled. Samples were immediately transported on ice to the laboratory for testing. Data on demographic characteristics for each livestock attendant was collected using a semi-structured questionnaire.

### Identification of *S. aureus* and Coagulase Negative Staphylococci

Pre-enrichment was carried out by incubating samples in Mueller–Hinton broth with 6.5% NaCl for 48 h at 37 °C. A volume of 10 µl of each sample was then plated on Mannitol salt agar and incubated for 48 h at 37 °C. Based on the colony morphology and ability to ferment mannitol, presumptive colonies were streaked on 5% sheep blood agar (Oxoid Ltd., Basingstoke, UK), and incubated for 24 h at 37 °C. Identification of staphylococci was achieved by using the matrix- assisted laser desorption ionization–time of flight mass spectrometry (MALDI-TOF MS) instrument. Briefly, the procedure for MALDI-TOF MS involved spreading well isolated colonies from overnight cultures on a steel target plate. This film is then overlaid with 1 µl of formic acid and allowed to dry for 15 min. 1 µl of matrix preparation containing cyano-4- hydroxy-cinnamic acid in 50% acetonitrile with 2.5% trifluoroacetic acid was placed on each sample and left to dry for a further 15 min. MALDI-TOF MS was then conducted and Ionization peaks generated were matched against the integrated reference library for speciation of the bacteria [60, 61].

### Antimicrobial susceptibility testing of staphylococci

Antimicrobial susceptibility testing was performed using Kirby Bauer’s disk diffusion method with 10 antimicrobial agents: (cefoxitin (30 µg), amikacin (30 µg), penicillin (1 unit), rifampicin (5 µg), clindamycin (2 µg), erythromycin (15 µg), gentamycin (10 µg), sulphamethoxazole/trimethoprim (25 µg), tetracycline (30 µg), linezolid (10 µg). The measured diameters of the zones of inhibition were interpreted according to the European Committee on Antimicrobial Susceptibility Testing (EUCAST) guidelines [62]. The minimum inhibitory concentration of cefoxitin-resistant isolate was done using vancomycin using E- test strips (bioMerieux) and interpreted based on EUCAST guidelines. Multidrug resistance was defined as resistance to three or more antimicrobial agents [63].

### Whole-genome sequencing and Analysis

Whole-genome sequencing was performed on all cefoxitin-resistant isolates using the illumina Miseq platform. DNA of freshly cultured isolates was extracted using Qiagen DNA MiniAmp kit following the manufacturer’s instructions. Extracted DNA was quantified using Qubit 4.0 Fluorometer assay kit (Thermo Fisher Scientific, MA), followed by library preparation with illumina DNA prep following the manufacturer’s instructions. The quality and concentration of fragmented DNA were assessed with the 2100 bioanalyzer system (Agilent) and qPCR (Kapa Sybr Fast qPCR kit) respectively. Libraries were then pooled and loaded on illumina 2 × 300 cycle cartridge for sequencing on Miseq platform (Illumina Inc., San Diego, CA).

Raw sequenced reads were quality filtered and trimmed using FASTQC (http://www.bioinformaticsbabraham.ac.uk/projects/fastqc/) and Trimmomatic ( http://www.usadellab.org/cms/index.php?page=trimmomatic) respectively with a minimum quality set at Q20 [64, 65]. Trimmed reads were de-novo assembled with Unicycler V0.4.9 from which only contigs greater than 200 bp were used for further analysis.

Assembled files were uploaded to Resfinder (https://cge.cbs.dtu.dk/services/ResFinder/), a tool available on Center for Genomic Epidemiology platform to detect resistance genes present in the sequenced isolates using an identity threshold of 90% and a minimum length of 60%. Plasmids and mobile genetic elements were predicted using Plasmidfinder (https://cge.cbs.dtu.dk/services/PlasmidFinder/) and MGEfinder (https://cge.cbs.dtu.dk/services/MobileElementFinder/) respectively. The sequence types of the isolates were predicted using MLSTFinder (https://cge.cbs.dtu.dk/services/MLST/).

Assembled sequences were mapped to a reference genome (GenBank accession number LS483305.1) and a core maximum likelihood phylogenetic tree was constructed using CSI phylogeny tool on Center for Genomic Epidemiology (CGE). Analysis was performed with default parameters. The resultant tree was annotated in the Interactive Tree of Life (iTOL) [66].

## Data Availability

The data sets used during the current study are available from the corresponding author on reasonable request.

## Abbreviations

AMR: Antimicrobial Resistance
CoNS: Coagulase Negative Staphylococci
EUCAST: European Committee on Antimicrobial Susceptibility Testing
MRSA: Methicillin resistant staphylococcus aureus
MALDI-TOF MS: Matrix-Assisted Laser Desorption/Ionization-Time of Flight *Mass Spectrometry*
MDR: Multi-drug resistance
MRS: Methicillin Resistant Staphylococcus
